# Diffusion-weighted image analysis along the perivascular space (DWI–ALPS) for evaluating interstitial fluid status: age dependence in normal subjects

**DOI:** 10.1007/s11604-022-01275-0

**Published:** 2022-04-27

**Authors:** Toshiaki Taoka, Rintaro Ito, Rei Nakamichi, Toshiki Nakane, Mayuko Sakai, Kazushige Ichikawa, Hisashi Kawai, Shinji Naganawa

**Affiliations:** 1 Department of Innovative Biomedical Visualization (iBMV), Nagoya University Graduate School of Medicine, 65 Tsurumai-cho, Showa-ku, Nagoya, Aichi 466-8550 Japan; 2 Department of Radiology, Nagoya University, Nagoya, Aichi Japan; 3 Canon Medical Systems Corporation, Otawara, Japan; 4 Department of Radiological Technology, Nagoya University Hospital, Nagoya, Aichi Japan; 5 Department of Radiology, Aichi Medical University, Nagakute, Japan

**Keywords:** DWI–ALPS method, DTI–ALPS method, Glymphatic system, MRI, Diffusion image

## Abstract

**Purpose:**

The purpose of this study was to evaluate the interstitial fluid status in a wide range of age groups using diffusion-weighted image analysis along the perivascular space (DWI–ALPS) method, which is a simplified variation of diffusion tensor image analysis along the perivascular space (DTI–ALPS).

**Materials and methods:**

This retrospective study included data from 128 patients who underwent clinical magnetic resonance imaging (MRI) studies, including DWI, and were found to have no abnormal findings in the brain on MRI. Three motion-probing gradients of the DWI were applied in an orthogonal direction to the imaging plane. Apparent diffusion coefficient images in the *x-*, *y-*, and *z-*axes were retrospectively generated, and composite color images were created to locate the projection and association fiber area on the slice including the body of the lateral ventricle. ALPS indices were calculated, and correlations with age were evaluated using linear and second-degree regression analysis. Linear regression analysis was also performed for a subgroup of patients older than 40 years. In addition, an analysis of variance (ANOVA) test among the generations was performed.

**Results:**

The linear regression analysis between age and the ALPS index showed a correlation coefficient of −0.20 for all age group and −0.51 for the subgroup older than 40 years. The second-degree regression analysis showed a correlation coefficient of 0.39. ANOVA showed that the 40’s generation showed a statistically significant higher value of ALPS index compared to all other generations except for the 30’s generation. While, the 70’s generation showed a statistically significant lower value of the ALPS index compared to all other generations.

**Conclusions:**

The analysis of the DWI–APLS method showed a correlation between age and the ALPS index in second-degree distribution which peaked in the 40’s generation. This finding in normal subjects may be fundamental in the analysis of disease cases.

**Secondary abstract:**

We tried to evaluate the glymphatic system status in a wide range of age groups using diffusion-weighted image analysis along the perivascular space (DWI–ALPS) method, and the results showed a correlation between age and the ALPS index in second-degree distribution which peaked in the 40’s generation.

## Introduction

The glymphatic system is a system for waste drainage via the cerebrospinal fluid or interstitial fluid along the perivascular space of the brain. In animal experiments, the activity of the glymphatic system was evaluated by intrathecal administration of tracers [1]. A tracer study of mice using fluorescent material and amyloid-β has been reported to show that the exchange efficiency between the subarachnoid cerebrospinal fluid (CSF) and brain parenchyma decreased dramatically with aging [2]. Another experimental study has also shown that the lymphatic function of the meninges is significantly reduced in aging mice, which may contribute to the age-related decline in cognitive function [3]. Several studies have evaluated glymphatic function in human subjects by intrathecal administration of gadolinium-based contrast agent (GBCA) tracers [4]. A tracer study using intrathecally administered GBCA to evaluate the effect of aging has also been reported and showed that the glymphatic pathway and meningeal lymphatic vessels might be impaired in aging humans. However, intrathecal administration of GBCA is an invasive method and has not been approved for clinical use.

As a non-invasive method to evaluate glymphatic function using diffusion tensor imaging (DTI), the “diffusion tensor image analysis along the perivascular space (DTI-ALPS)” technique has been developed [5]. In this method, ALPS index, which is the ratio of water diffusivity along the perivascular space, is used to indicate glymphatic function, and many publications have demonstrated glymphatic insufficiency in various pathologies using this method [6–29]. ALPS index has been reported to be reduced in Alzheimer's disease cases [5, 10]. In the detection of in vivo pathological hallmarks in Alzheimer’s disease, such as markers of amyloid burden, tau pathology and neurodegeneration, the ALPS method might be an imaging biomarker for amyloid burden or tau pathology. In addition to the evaluation of neurodegeneration with the morphological imaging, MRI with ALPS method might be a one-stop shop method for Alzheimer’s disease evaluation.

DTI is an imaging method that applies a motion-probing gradient (MPG) in at least six directions, but usually in 12–20 directions, and is not usually used in daily clinical practice. Instead, diffusion-weighted imaging (DWI), in which MPG is applied in three directions, is frequently used in clinical settings. In a study examining the reproducibility of the DTI–ALPS method (CHAMONIX study), the number of MPGs was also examined, and it was reported that MPG = 3, that is, the ALPS index by DWI and the ALPS index by DTI in 12 axes showed a good correlation [15].

In the current study, we developed a method to use DWI with a three-axis MPG to obtain the DWI–ALPS index. We evaluated the correlation between age and DWI–ALPS index in normal subjects using clinical DWI with acquisition times as short as 1 min 11 s. The purpose of this study was to evaluate the glymphatic system in a wide range of age groups using the DWI–ALPS method, to clarify the changes in the DWI–ALPS index with normal aging and to establish a standard for evaluating the DWI–ALPS index in future disease cases.

## Subjects and methods

This retrospective study was performed with the permission of the institutional review board of our institute (2021–2028). The study included the data of 128 patients who underwent clinical magnetic resonance imaging (MRI) studies including DWI from November 2020 to September 2021 and were found to have no abnormal findings in the brain on MRI. Patients with minor anomalies on MRI were also not enrolled in the study, including brain atrophy, ventricular enlargement, and leukoaraiosis. The subjects were limited to cases that were older than 10 years, in which myelination of the white matter was completed. The oldest subject in the current study was 84 years of age. The subjects included 60 male and 68 female. The reasons for the order of the imaging study on MRI were as follows headache: 23 cases; psychological abnormality: 32 cases; visual abnormality: 5cases; screening for metastasis and no metastasis found: 26 cases; screening for aneurysm and no aneurysm found: 38 cases; and follow-up study for aneurysms smaller than 3 mm: 4 cases (Table 1).

Images were acquired using 3 T clinical scanner (Vantage Centurian, Canon Medical Systems, Tochigi, Japan). The DWI included in the clinical sequence was as follows, echo-planar imaging: repetition time= 5000 ms, echo time= 85 ms, motion-probing gradient= 3 axes, *b* value= 1000 s/mm^2^, number of averages= 1 for *b* = 0 and 2 for *b* = 1000, acquisition time= 1 min 11 s, axial imaging plane on the anterior commissure–posterior commissure (AC–PC) line. The three MPGs were applied in an orthogonal direction to the imaging plane, not to the static magnetic field.

**Fig. 1:**
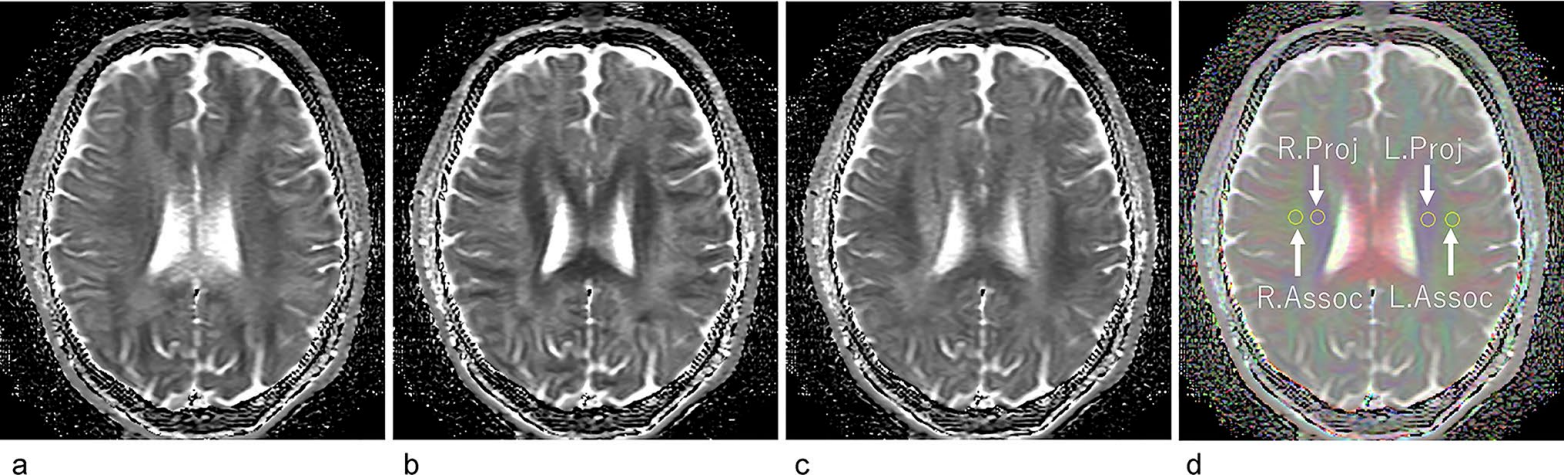
Calculation of the DWI–ALPS index. Apparent diffusion coefficient (ADC) images in the *x-*axis (**a**), *y-*axis (**b**), and *z-*axis (**c**) were retrospectively created from the b = 0 images and diffusion-weighted images (DWI) of the *x-*, *y-*, and *z-*axes. Color composite images were created as follows: *x* = red, *y* = green, and *z* = blue (**d**). On the slice including the body of the lateral ventricle of the composite color image (**d**), we identified the projection fiber area (R. Proj and L. Proj on **d**) and association fiber areas (R.Assoc and L. Assoc on **d**). For these ROIs, we measured ADC values along the *x-*, *y-*, and *z-*axes to calculate the DWI–ALPS index, which is given by the ratio of the mean of the *x-*axis ADC in the area of the projection fiber (ADCxproj) and the *x-*axis diffusivity in the area of association fibers (ADCxassoc) to the mean of the *y-*axis diffusivity in the area of projection fiber (ADCyproj) and *z-*axis diffusivity in the area of association fibers (ADCzassoc) as follows: DWI-ALPS index = mean (ADCxproj, ADCxassoc)/mean (ADCyproj, ADCzassoc)

We retrospectively generated apparent diffusion coefficient (ADC) images in the *x-*, *y-*, and *z-*axes (Fig. 1a–c) and created composite color images from the *b* = 0 images and the diffusion-weighted images for the *x-*, *y-*, and *z-*axes in the same manner as the color fractional anisotropy images of DTI, that is, *x* axis = red, *y* axis = green, and *z* axis = blue (Fig. 1d). On the slice including the body of the lateral ventricle of the composite color image, we identified the projection fiber and association fiber areas and measured ADC values along the *x-*, *y-*, and *z-*axes. It is known that the perivascular space runs along the *x-*axis in this area [5, 30–32]. We calculated the DWI–ALPS index, which is given by the ratio of the mean of the *x-*axis ADC in the area of the projection fiber (ADCxproj) and the *x-*axis ADC in the area of association fibers (ADCxassoc) to the mean of the *y-*axis ADC in the area of projection fiber (ADCyproj) and *z-*axis ADC in the area of association fibers (ADCzaccoc) as follows:

$${\text{DWI}}{-}{\text{ALPS index}} = {\text{mean }}\left( {{\text{ADCxproj}},{\text{ ADCxassoc}}} \right)/{\text{mean }}\left( {{\text{ADCyproj}},{\text{ ADCzassoc}}} \right)$$

Generation of ADC images, composite images, and calculation of the DWI–ALPS index were performed using ImageJ (version 1.50b) with a house-made macro script.

Region of interest (ROI) placement and measurements were performed independently by two neuroradiologists (TT and SN) using the ImageJ macro script. The correlation coefficients of the two observers' measurements were calculated for the DWI–ALPS index measured by the two observers. The final DWI–ALPS index for the evaluation with age was defined as the average value of the two observers' measurements. We performed a linear regression and a second-degree regression analysis between the DWI–ALPS index and age. Linear regression analysis was also performed for a subgroup of subjects older than 40 years. In addition, the value of the DWI–ALPS index in normal subjects was examined with age, and comparisons by age group by decades were made by analysis of variance (ANOVA) test. Statistical analysis and generation of graphs were performed using R (version 4.02).

## Results

**Fig. 2:**
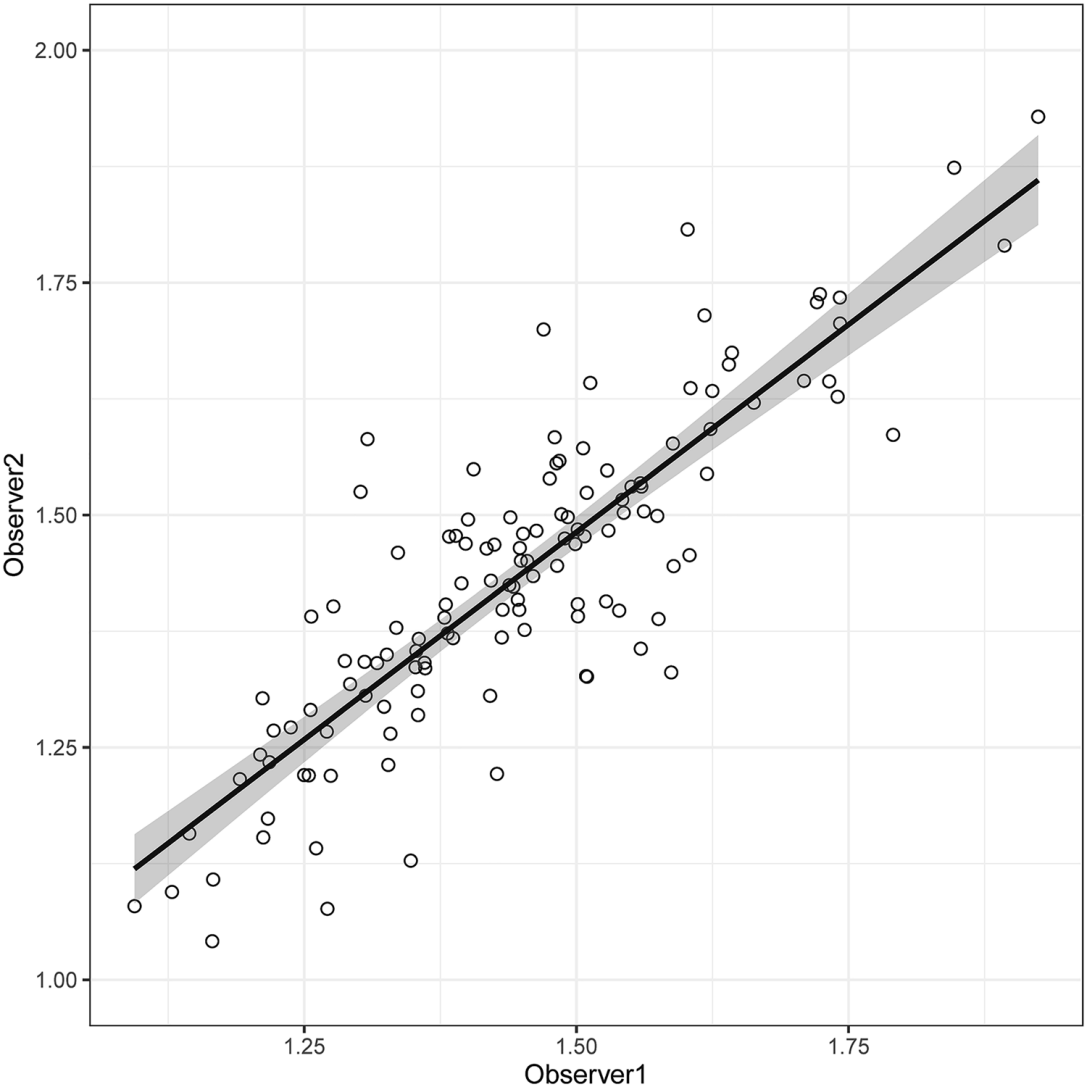
Correlation of the measured ALPS index by two independent observers. Regression lines and 95% CIs (gray areas) are shown. The correlation coefficient between the measurements from the two observers was 0.85

Figure 2 shows the correlation between two independent measurements of the DWI–ALPS index by the two observers with two-sided 95% confidence intervals (CI). The correlation coefficient between the measurements from the two observers was 0.85.

**Fig. 3:**
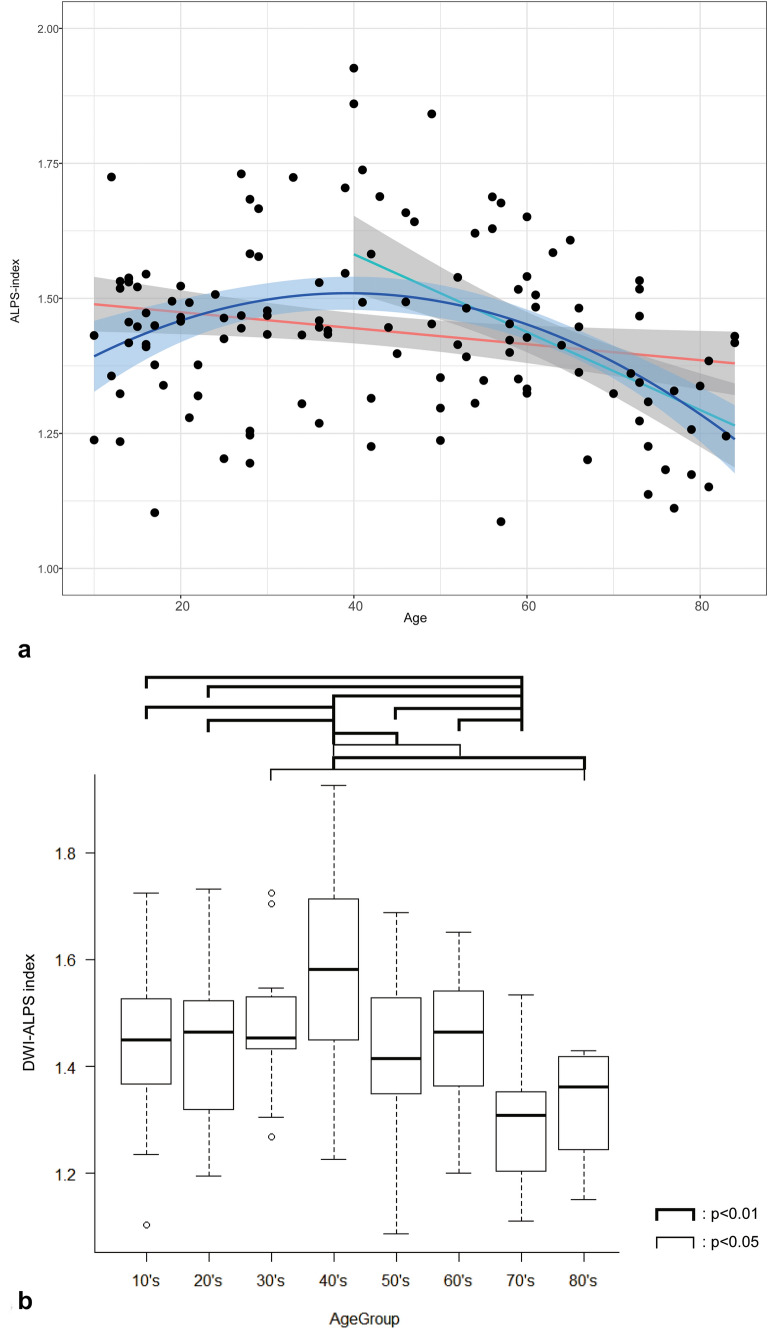
DWI–ALPS inde*x-*to-age plot in normal subjects. **a** is the DWI–ALPS inde*x-*to-age plot. The red line shows the linear regression line of the whole subject (*r* = −0.20), while the green line shows linear regression line of the subject older than 40 years (*r* = −0.51). The blue line shows the secondary regression line (*r* = 0.39). The 95% confidence intervals are shown as gray area. **b** Is the boxplot of the DWI–ALPS index of age groups (10’s to 80’s) including the minimum, lower quartile, median, upper quartile, and maximum, and open circles indicate outliers. The ANOVA test showed a statistically significant higher DWI-ALPS index in the age group of 40’s and lower index in the age group of 70–79

Figure 3 shows the scatter diagram of the age and DWI–ALPS index, which are the mean values obtained by the two observers. The linear regression line with a two-sided 95% CI is shown as a red line and the second-degree regression curve with a two-sided 95% CI are shown as a blue line. Linear regression analysis between age and the DWI–ALPS index showed a correlation coefficient of −0.20. The multiple correlation coefficient by the second-degree regression analysis was 0.39. Linear regression analysis for the subgroup of 69 subjects older than 40 years showed a correlation coefficient of −0.51.

The results of the ANOVA analysis over the decades are shown in Fig. 3. The 40’s generation showed a statistically significant higher value of the DWI–ALPS index compared to all other generations, except for the 30’s generation. Meanwhile, the 70’s generation showed a statistically significant lower value of the DWI–ALPS index compared to all other generations.

## Discussion

In the current study, DWI acquired in clinical MRI examinations in which MPGs were applied in the orthogonal direction were retrospectively utilized to calculate the DWI–ALPS index in subjects aged 10–84 years. The statistical evaluation showed reproducibility in the calculation of the DWI–ALPS index by two observers, with a correlation coefficient of 0.85. The second-degree regression showed a higher correlation compared to the linear regression for subjects of all age ranges, indicating that the distribution is more likely to have a peak than linear distribution. The peak is on the second-degree regression line in the subjects in their 40 s, which was supported by the result of the ANOVA by age group. Linear regression analysis for the subgroup older than 40 years of age showed a rather large correlation coefficient of −0.51.

More than 20 reports in which the ALPS method was applied to evaluate glymphatic system status have been published [5–26, 28]. Many reports have shown a negative correlation between age and ALPS index [5, 9, 11, 13, 14, 16, 28]. McKnight et al. showed that there was a negative correlation between the ALPS index and age, with a Spearman’s rank correlation coefficient of −0.203 in the group of subjects with Parkinson’s disease or essential tremor approximately ranging from 40 to 80 years [13]. Zhang et al. showed a negative correlation between age and the ALPS index with a correlation coefficient of − 0.209 in 142 normal elderly subjects older than 50 years [14]. Ma et al. showed a negative correlation between age and the ALPS index with a standard partial regression coefficient of − 0.012 in patients with Parkinson’s disease [22]. Siow et al. also showed a negative correlation between age and the ALPS index with a standard partial regression coefficient of − 0.035 in communit*y-*dwelling older adults older than 60 years [23]. In a study of the younger generation, Lee et al. showed a negative correlation between age and ALPS index with a correlation coefficient of −0.375 in the group of patients with juvenile myoclonic epilepsy ranging from 12 to 46 years [16]. Toh et al. examined cases of glioma with an age range of 18–91 years. Although the correlation coefficient was as low as −0.147, the graph indicating a correlation between age and the ALPS index seems to have a slight peak around 40 years [17].

The above observation of the negative correlation between age and the ALPS index seems to be consistent with the findings of the tracer study. An experimental study using radiotracer clearance assays in young, middle-aged, and old mice showed that advancing age was associated with a dramatic decline in the efficiency of exchange between the subarachnoid CSF and the brain parenchyma [2]. Another experiment using a mouse model of multiple microinfarcts induced by intra-arterial injection of cholesterol crystals showed that multiple microinfarcts markedly impaired the global influx of CSF along the glymphatic pathway. The effect of diffuse microinfarcts on global glymphatic pathway function was exacerbated in the old mice aged 12 months compared with that in 2–3-month-old young mice [33]. In a previous study, intrathecal administration of the gadolinium-based contrast agent (GBCA) was performed in human subjects. In this study, signal changes in the brain after intrathecal GBCA administration were observed as an indicator of glymphatic function and revealed that the clearance of the glymphatic pathway was related to aging [34]. In the figures presented in the report, indices for glymphatic clearance tended to decline after the age of 40 years. There are several tracer studies on intravenous GBCA administration in human subjects [35, 36]. One study observed GBCA leaks from the cortical veins into the surrounding CSF and showed that GBCA leakage from the cortical veins was seen in older subjects (> 37 years), but not in the five younger subjects (younger than 37 years) [36]. These results suggest that glymphatic function tends to decline after approximately 40 years of age compared to older or younger age groups.

For the findings after the age of 40, the results of the current study are consistent with the previous studies mentioned above on the relation between glymphatic function and age, in that our results also showed a decline in the DWI–ALPS index. One of previous studies showed the decline in glymphatic CSF–ISF lead to the accumulation of tau in the brain in Alzheimer's disease model mice [37]. Another previous animal study suggested that an age-related reduction in C–C chemokine receptor type 7 dependent decline of immune cells through the lymphatic vasculature mediates brain aging and potentially exacerbates cognitive decline and brain amyloid-β pathology [38]. In human, several in-vivo study using ALPS method showed age related decline in the normal elderly population [14, 23] or in the cases of Parkinson’s disease [13, 22]. In contrast to the current results in the elderly, the results of our study differed from those of previous studies in that the index peaked in the 40 s. It should be noted that the data presented here is only the relationship between the DWI–ALPS index and age, and there is no guarantee that the lymphatic system and age have this relationship. Because the ALPS index is calculated from diffusion images, it is necessary to consider the possible influence of age on the state of white matter fibers. In a study on the effect of aging on ADC for the age range of 20–89 years, the ADC values gradually increased with aging [39]. In another study on age-related changes in brain white matter anisotropy, in the population of 23–76 years, the fractional anisotropy of the white matter showed a linear decline across all age groups and there was no peak [40]. On the other hand, in a report on healthy individuals aged 18–94 years, fractional anisotropy of the white matter showed a linear decline; however, the result of the diffusivity showed a negative peak at the age of 40 in the report [41]. We cannot deny the possibility that age-related changes in the state of diffusion in the brain may at least partially affect the ALPS index. Therefore, the phenomenon that the ALPS index peaked in the 40 s in the present data should be used as a matter of caution when evaluating the ALPS index at each age.

In children, the blood–brain barrier is underdeveloped, and as they grow, the function of the blood–brain barrier develops [42, 43]. The finding in the current study that the ALPS index was higher in the middle-aged than in the young in this study may reflect the developmental process of the interstitial circumstance in the brain. The relationship between age and interstitial fluid dynamics, including the waste excretion system in the brain, is still unclear in many respects. There have been reports of diminished integration of the blood–brain barrier with age [44–46]. However, there is a lack of research on the degree of development of the blood–brain barrier in humans from a young age, and there is no clear answer as to the age at which the function of the blood–brain barrier peaks. Some previous studies support the peak in the middle-aged generation found in this study. In a study of human subjects that examined the relationship between age and P-glycoprotein, an efflux transporter from the blood–brain barrier, as an indicator of blood–brain barrier function, blood–brain barrier function was higher in the middle-aged group than in the younger and older age groups, forming an age-related peak [47].

There were several limitations to the current study. First, this was a retrospective study of routine clinical images. Although all DWIs were acquired in the AC–PC line, the head posture was not unified. In the reproducibility study of the DTI–ALPS method, modification of the head position impaired reproducibility of the ALPS index [15], which may have led to variations in the ALPS index. Second, the number of subjects was insufficient. The number of subjects included in each decade was 6 to 21, which may lead to less statistical power. Third, since this study is retrospective in nature, the background of the subjects varied widely according to age group. For example, psychological abnormality was dominant in the younger generation and screening for metastasis was dominant in the older generation, which will affect the results of this study. Another limitation is the lack of cognitive function data in the cases included in this study. However, this is also a fact that indicates that the attending physicians did not determined that cognitive function tests were necessary based on clinical symptoms.

## Conclusions

The ALPS index which is likely to indicate the status of interstitial fluid dynamics in the brain or the glymphatic function can be calculated using DWI used in clinical studies. Our results showed a correlation between age and the ALPS index in second-degree distribution which peaked in the 40’s generation. While DWI requires a shorter imaging time and is widely used in clinical practice, the DWI–ALPS method seems to be useful for evaluating glymphatic function in a wide variety of clinical cases with short acquisition time. This finding in normal subjects would be fundamental in the analysis of disease cases in the future.

**Table 1:** The distribution of the subjects

Generation	Male	Female	Sub total	The reasons for the order of the imaging study					
				Headache	Psychological abnormality	Visual abnormality	Screening for metastasis	Screening for aneurysm	Follow-up for small aneurysm (< 3 mm)
10**–**19	11	13	24	13	10	1			
20**–**29	9	12	21	7	13	1			
30**–**39	6	8	14	2	7	2		3	
40**–**49	7	8	15		1		2	11	1
50**–**59	9	10	19	1	1		8	8	1
60**–**69	8	6	14				8	5	1
70**–**79	7	8	15				5	9	1
80**–**89	3	3	6			1	3	2	
